# The Role of *PNPLA3*_rs738409 Gene Variant, Lifestyle Factors, and Bioactive Compounds in Nonalcoholic Fatty Liver Disease: A Population-Based and Molecular Approach towards Healthy Nutrition

**DOI:** 10.3390/nu16081239

**Published:** 2024-04-21

**Authors:** Meiling Liu, Sunmin Park

**Affiliations:** 1 Department of Chemical Engineering, Shanxi Institute of Science and Technology, Jincheng 048000, China; liumeiling@sxist.edu.cn; 2 Department of Bioconvergence, Hoseo University, Asan 31499, Republic of Korea; 3 Department of Food and Nutrition, Institute of Basic Science, Obesity/Diabetes Research Center, Hoseo University, Asan 31499, Republic of Korea

**Keywords:** NAFLD, *PNPLA3*, AMPK, *SREBP-1C*, HepG2, compound C

## Abstract

This study aimed to investigate the impact of a common non-synonymous gene variant (C>G, rs738409) in patatin-like phospholipase domain-containing 3 (*PNPLA3*), leading to the substitution of isoleucine with methionine at position 148 (*PNPLA3*-I148M), on susceptibility to nonalcoholic fatty liver disease (NAFLD) and explore potential therapeutic nutritional strategies targeting *PNPLA3*. It contributed to understanding sustainable dietary practices for managing NAFLD, recently referred to as metabolic-dysfunction-associated fatty liver. NAFLD had been diagnosed by ultrasound in a metropolitan hospital-based cohort comprising 58,701 middle-aged and older Korean individuals, identifying 2089 NAFLD patients. The interaction between *PNPLA3* and lifestyle factors was investigated. In silico analyses, including virtual screening, molecular docking, and molecular dynamics simulations, were conducted to identify bioactive compounds from foods targeting *PNPLA3*(I148M). Subsequent cellular experiments involved treating oleic acid (OA)-exposed HepG2 cells with selected bioactive compounds, both in the absence and presence of compound C (AMPK inhibitor), targeting *PNPLA3* expression. Carriers of the risk allele *PNPLA3*_rs738409G showed an increased association with NAFLD risk, particularly with adherence to a plant-based diet, avoidance of a Western-style diet, and smoking. Delphinidin 3-caffeoyl-glucoside, pyranocyanin A, delta-viniferin, kaempferol-7-glucoside, and petunidin 3-rutinoside emerged as potential binders to the active site residues of *PNPLA3*, exhibiting a reduction in binding energy. These compounds demonstrated a dose-dependent reduction in intracellular triglyceride and lipid peroxide levels in HepG2 cells, while pretreatment with compound C showed the opposite trend. Kaempferol-7-glucoside and petunidin-3-rutinoside showed potential as inhibitors of *PNPLA3* expression by enhancing AMPK activity, ultimately reducing intrahepatic lipogenesis. In conclusion, there is potential for plant-based diets and specific bioactive compounds to promote sustainable dietary practices to mitigate NAFLD risk, especially in individuals with genetic predispositions.

## 1. Introduction

Nonalcoholic fatty liver disease (NAFLD), a prevalent medical condition characterized by hepatic fat accumulation, liver inflammation, fibrosis, cirrhosis, and the potential to progress to liver cancer [1], has recently been redefined as metabolic-dysfunction-associated fatty liver disease (MAFLD) to reflect its multifactorial nature better and encompass a broader spectrum of liver disorders [2]. While NAFLD and MAFLD share many similarities, including their clinical manifestations and risk factors, they somewhat differ [2]. We have chosen to adhere to the NAFLD definition and terminology. NAFLD affects approximately 15 to 30% of adults globally, highlighting its significant public health impact [3]. While ultrasound scans offer a non-invasive means of detecting liver fat, a liver biopsy remains the gold standard for accurate diagnosis, particularly in cases of mild NAFLD [4]. Despite conducting multiple studies and clinical trials using various treatments, including weight loss drugs, insulin sensitizers, lipid-lowering drugs, and antioxidants such as vitamins C and E, none of them have shown definite efficacy for NAFLD. Recently, the US FDA has approved the drug Rezdiffra (resmetirom) in combination with diet and exercise for the treatment of adult NAFLD/non-cirrhotic nonalcoholic steatohepatitis (NASH) [5]. Certain phytochemicals, including flavonoids, show promise due to their antioxidative properties and ability to improve insulin sensitivity and regulate lipid metabolism, suggesting a potential role in preventing or slowing the progression of NAFLD. Studies have highlighted the ability of some flavonoids to modulate glucose and lipid metabolism, shield the liver, and ameliorate fatty liver. However, the precise mechanisms of action remain incompletely understood [6,7,8,9]. Further exploration of medications for NAFLD is warranted.

Understanding the genetic and environmental factors influencing NAFLD development is crucial for effective prevention and treatment with targeted interventions for at-risk individuals. A critical aspect of this interplay between genetic and environmental factors involves AMP-activated protein kinase (AMPK), a pivotal regulator of metabolic pathways that inhibits hepatic lipid synthesis and promotes lipid oxidation, effectively balancing lipid metabolism [10]. Moreover, recent studies have revealed a significant association between the patatin-like phospholipase domain-containing protein-3 (*PNPLA3*)_rs738409C/G polymorphism and NAFLD, highlighting its potential impact on liver fat metabolism [11]. Several studies have shown that *PNPLA3* overexpression induces hepatic steatosis through the carbohydrate response element binding protein (*ChREBP*) and sterol regulatory element binding protein 1c (*SREBP1c*), and its silencing prevents the development of hepatic fat storage and inflammation, thereby effectively preventing the development of NAFLD [12,13,14]. Furthermore, hepatic lipogenesis is linked to the AMPK pathway, which can modulate *PNPLA3*. However, it remains unclear whether AMPK activation decreases *PNPLA3* expression in the liver and prevents NAFLD and whether the decreased binding energy of bioactive compounds with *PNPLA3* reduces its expression to reduce hepatic lipogenesis.

This study aimed to find a sustainable diet to prevent and mitigate NAFLD in individuals with a genetic predisposition. We explored genetic variants that affect NAFLD risk and their interaction with lifestyle factors, such as nutritional intake, in the middle-aged and elderly individuals of the Korea Genome and Epidemiology Study (KoGES) cohort. Furthermore, we employed a molecular docking system for the *PNPLA3*_rs738409C/G protein to identify bioactive compounds that could potentially interact with the protein. The molecular mechanism of the identified bioactive compounds was further examined in a cell-based experiment using HepG2 cells. By combining epidemiological data, molecular docking, and cell-based experiments, this study contributed to the development of personalized dietary interventions for individuals with a genetic predisposition to NAFLD, ultimately contributing to the prevention and mitigation of the disease.

## 2. Methods

### 2.1. Participants

Between 2004 and 2013, 58,701 participants (20,293 males and 38,408 females) voluntarily participated in the hospital-based urban cohort as part of the Korea Genome and Epidemiology Study (KoGES). The sample size was determined using the Gpower calculator, achieving significance at α = 0.05, β = 0.99, with an odds ratio of 1.06 between NAFLD and healthy participants. With over 50,000 participants, this goal was achieved. This study followed the Declaration of Helsinki and was approved by the Institutional Review Boards of the Korea National Institutes of Health (KBP-2019-055) and Hoseo University (1041231-190902-BR-099-01). Every participant completed a written informed consent form.

### 2.2. Basic Characteristics of the Participants and Biochemical Measurements

Participants were interviewed and queried about their demographics, lifestyle habits, and health status, including their gender, age, education, and income. Household income was divided into three levels ($/month): low income ($2000), middle income ($2000–$4000) and high income ($4000) [15]. The participants were classified into smokers and non-smokers. Those who smoked > 100 cigarettes in the 6 months prior to the participation in the study were considered smokers [16]. Regular physical activity was defined as 150 min per week of moderate physical activity (e.g., mowing, swimming, badminton, or tennis). Anthropometric measurements and biochemical assays of the serum were carried out. Body weight, height, and waist circumference were assessed using established methods [17]. Blood was drawn from the subjects after fasting for more than 12 h. Plasma and serum were separated from the collected blood after centrifugation, and glucose and lipid profiles were assessed in the plasma and serum samples, respectively [18]. Serum total cholesterol, high-density lipoprotein cholesterol (HDL-C), triglycerides, aspartate aminotransferase (AST), and alanine transaminase (ALT) concentrations were measured using an enzyme-linked immunosorbent assay (ELISA) kit (Asan Pharm., Seoul, Republic of Korea).

### 2.3. Food and Nutrient Intake and Dietary Pattern Using a Semi-Quantitative Food Frequency Questionnaire (SQFFQ)

Dietary habits were assessed using an SQFFQ. Over the previous 12 months, participants reported their typical food intake based on 106 items commonly consumed in the Korean diet. This SQFFQ was validated against four seasons of three-day food records [19,20]. Food intake was determined by multiplying the frequency of consumption for each food by the daily amount, as previously described, and expressed in grams per day. The daily consumption of energy, carbohydrates, fats, proteins, vitamins, and minerals was computed from SQFFQ data using the computer-aided nutritional analysis program CAN-Pro 2.0, developed by the Korean Nutrition Society.

The food items in the SQFFQ were categorized into 30 predetermined food groups, which were utilized in constructing dietary patterns through principal component analysis (PCA). Four distinct dietary patterns were identified by applying eigenvalues greater than 1.5 and employing orthogonal rotation (varimax). The predominant contributing foods in each dietary pattern were assigned factor-loading values of ≥0.40. The identified groups were labeled as the Korean balanced diet (KBD), plant-based diet (PBD), Western-style diet (WSD), or rice-based diet (RBD) groups.

### 2.4. Definition of NAFLD

Since heavy drinking (30 and 20 g of alcohol or higher per day in males and females, respectively) could also induce fatty liver disease, heavy drinkers were excluded. The participants who were diagnosed with NAFLD by a physician with an ultrasound method and had serum AST concentrations ≥ 40 U/L and ALT ≥ 35 U/L were regarded as a risk group (*n* = 2089). The participants with no NAFLD diagnosis and serum AST concentrations < 40 U/L and ALT < 35 U/L were considered normal levels (*n* = 48,999).

### 2.5. Genotyping and Quality Control

DNA was isolated from the peripheral blood of the participants, and genotyping was conducted using the Affymetrix Genome-Wide Human single-nucleotide polymorphism (SNP) Array 5.0 (Affymetrix, Santa Clara, CA, USA). Genotyping quality and accuracy were determined using the Bayesian robust linear model with the Mahalanobis distance classifier (BRLMM) genotyping algorithm [18]. The exclusion criteria for DNA genotyping were as follows: high genotype deletion detection rate (≥4%), low genotyping accuracy (<98%), high heterozygosity (>30%), minor allele frequency (MAF) < 0.01, Hardy–Weinberg equilibrium (HWE) (*p* < 0.05), and having gender bias [18].

### 2.6. Genetic Variants Affecting NAFLD in Koreans

GWAS analysis explored and identified susceptible genetic variants that increase NAFLD risk. Single-nucleotide polymorphisms (SNPs) influencing NAFLD risk have been identified using PLINK in a Korean population. The age, gender, body mass index (BMI), residential area, physical activity, education, smoking, and energy intake of the participants were used as covariates in the GWAS analysis. The gene names for the SNPs were found by examining the g:Profiler annotation database.

### 2.7. Screening of Bioactive Compounds in Foods to Have Low Binding Energy with PNPLA3

We first extracted the *PNPLA3* protein sequence in the Fasta format from UniProt (ID: Q9NST1; https://www.uniprot.org/uniprot/Q9NST1, 7 July 2022). Subsequently, we obtained the 5FYA protein using SWISS-MODEL homology modeling. The protein structure was prepared to enhance the protein structure using the Discovery Studio software version 4.5 package. It involved hydrogenating the protein structure, completing missing amino acids, and removing metal ions and water molecules. Additionally, we used the Swiss-PdbViewer to optimize the protein’s energy.

We converted 22,589 compounds from food received from Foodb to the protein data bank, partial charge (q), and atom type (t) (pdbqt) format using Open Babel v3.1.0. The Foodb website includes a freely accessible database providing detailed information on the chemical composition of typical unprocessed foods, including micronutrients and macronutrients. The DoGSiteScorer online tool (https://proteins.plus/, 30 July 2022) was employed to predict and elucidate potential binding pockets in the wild and mutated types of *PNPLA3* (I148M) crystals. We examined four molecular dynamics systems using Discover Studio software (Biovia, Discovery Studio Visualizer, software version 20.1.0.19295) to evaluate binding pocket characteristics such as size, surface area, and drug ability score to generate the wild-type *PNPLA3* (WT), mutant *PNPLA3* (MU), *PNPLA3* binding with a small molecule (ligand), and *PNPLA3* mutant binding with a small molecule (ligand-MU). We referred to the CHARMM36 force field parameter table in the Discovery Studio software to define the simulation parameters for the small molecules. The Discovery Studio Visualizer also generated visualizations of the protein–ligand binding structures in both 2D and 3D.

### 2.8. Molecular Dynamics Simulation (MDS)

MDS is a promising method to examine the conformational changes in the structure of Ile148Met variants relative to the native conformation [21,22]. MDS can detect changes in protein phenotypes, which helps validate the severe implications of disease-associated mutations predicted computationally [23]. MDS of the *PNPLA3*–compound complex was performed using the DS/Standard Dynamics Cascade protocol; the molecule was solvated under periodic boundary conditions, and the CHARMM36 force field was added. The ‘Standard Dynamics Cascade’ approach was implemented to set the parameters for the MDS of the *PNPLA3* within the solvent system. The default values were used for most parameters, with specific settings: a ramp time of 40 ps, a balancing time of 400 ps, a simulation sampling time of 10,000 ps, and a simulation step size of 2 fs. Various analyses were performed after conducting the simulation for a duration of 10 ns. The root mean square deviation (RMSD), root mean square fluctuation (RMSF), and hydrogen bond values were analyzed to investigate the stability and dynamics of the system.

### 2.9. Cell Culture

HepG2 cells were cultured in a high-glucose Dulbecco’s Modified Eagle Medium (DMEM) and placed in a 37 °C, 5% CO_2_ incubator. Oleic acid (OA) was used to simulate in vitro hepatic steatosis. HepG2 cells were exposed to different doses of OA (0, 0.1, 0.3, 0.5, 0.7, 1, 1.5, 2, and 3 mM), the five bioactive compounds (20, 50, 70, 100, 150, and 200 μM), and compound C (AMPK inhibitor). Cell viability was assessed using the 3-(4,5-dimethylthiazol-2-yl)-2,5-diphenyltetrazolium bromide (MTT) assay, which measures cell viability by detecting the conversion of MTT to a purple formazan dye, and the color change was measured at 550 nm. The levels of lipid peroxides in the cells were determined using a thiobarbituric acid reactive substances (TBARS) kit (DoGenBio, Seoul, Republic of Korea).

### 2.10. Realtime PCR

Total RNA was extracted from the cells using a phenol and guanidine isothiocyanate-based single-phase solution (Trizol reagent, Invitrogen, Rockville, MD, USA). The cDNA synthesis was performed by combining equal amounts of total RNA, superscript III reverse transcriptase, and high-fidelity Taq DNA polymerase. The cDNA was utilized for the polymerase chain reaction (PCR) with the corresponding primers in a real-time PCR machine (BioRad Laboratories, Hercules, CA, USA) to measure the mRNA expression of *PNPLA3* and sterol regulatory element binding protein 1c (*SREBP-1c*) genes. The primers are listed in Supplementary Table S1. The gene expression levels in each sample were quantified using the threshold comparison cycle (CT) method after amplifying the expression by a real-time polymerase chain reaction.

### 2.11. Statistical Analysis

Statistical analysis was performed using the SPSS software version 21 (IBM Inc., Armonk, NY, USA). All results were presented as mean ± standard deviation. In a population study, the odds ratios (ORs) and 95% confidence intervals (CIs) of NAFLD risk with the biochemical parameters were evaluated using a logistic regression analysis after adjustment for covariates such as age, gender, body mass index (BMI), residential area, physical activity, education, smoking, and energy intake. The interaction between the selected genetic variant for NAFLD risk and lifestyles was evaluated in a two-way analysis of covariance (ANCOVA) while adjusting for the mentioned covariates. Univariate analysis of variance is employed in cellular research to compare the variables associated with metabolic changes among the bioactive compound groups and control group. Multiple groups were analyzed using Tukey’s test, with a significance level set at *p* < 0.05.

## 3. Results

### 3.1. Basic Characteristics of the Participants

The hospital-based urban cohort consisted of 51,088 participants, out of which 2089 individuals (4%) were diagnosed with NAFLD. Upon adjusting for other factors, women exhibited a 1.3-fold higher incidence of NAFLD than men (Table 1). BMI and waist circumference were significantly associated with NAFLD risk, with 2.6-fold and 1.5-fold higher risks, respectively. Individuals with NAFLD had higher plasma total cholesterol and triglyceride concentrations than those without NAFLD (Table 1). Moreover, the incidence of hypertension and type 2 diabetes in the NAFLD group was 1.3 and 2.3 times higher, respectively, than in the non-NAFLD group. Alcohol consumption and smoking were also more prevalent among the NAFLD group as opposed to the non-NAFLD group. However, there were no significant differences in age, education, income, or dietary nutrient intake between the non-NAFLD and NAFLD groups (Table 1).

### 3.2. Genetic Variations Associated with NAFLD Risk

GWAS was used to investigate the genetic variations linked to NAFLD risk. The genetic variant found to be significantly associated with NAFLD was *PNPLA3*_rs738409. The adjusted odds ratio (OR) was 1.487, indicating that the risk of NAFLD with the minor allele of the SNP was 1.487 times higher than with the major allele (Table 2). The minor allele of *PNPLA3*_rs738409 was thus the risk allele.

### 3.3. Interaction of PNPLA3_rs738409 with Lifestyles

The interaction of *PNPLA3*_rs738409 with smoking significantly affected NAFLD risk. Both major and minor *PNPLA3*_rs738409 alleles were positively associated with NAFLD risk in smokers and non-smokers. However, their association was much higher in former and current smokers (3.12-fold) than in non-smokers (1.89-fold) (Table 3). Among the four dietary patterns (Korean balanced diet, plant-based diet, Western-style diet, and rice-main diet), the plant-based diet (PBD) and Western-style diet (WSD) interacted with *PNPLA3_rs738409*. A PBD was inversely associated with NAFLD risk, but a WSD was positively linked (Table 3). The smoking status also interacted with the NAFLD risk in participants carrying *PNPLA3*_rs738409 minor alleles. However, the interaction between exercise and *PNPLA3*_rs738409 had no significant impact on the risk of NAFLD (Table 3). For participants carrying *PNPLA3*_rs738409 minor alleles, there may also be an increased risk of NAFLD with a low PBD, a high WSD, and smoking.

### 3.4. Molecular Docking

**Figure 1:**
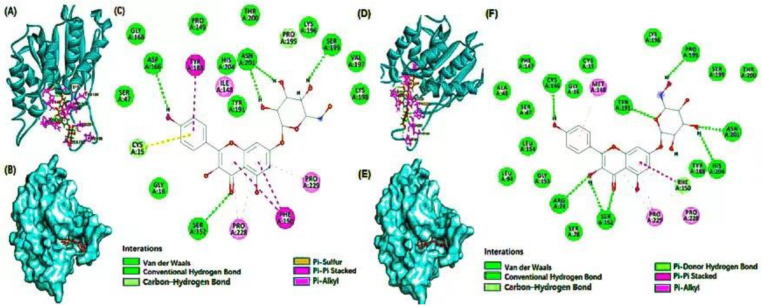
Molecular docking of *PNPLA3* WT (I148) and MT (148M) with kaempferol-7-glucoside. Molecular docking with the bioactive compound and *PNPLA3* wild type (WT, I148) and mutated type (MT; 148M). (**A**,**D**) Diagrammatic representation of the compound (ball and stick model) binding with WT and MT of *PNPLA3*, respectively (**B**,**E**) binding of the compound at the central cavity with WT and MT of *PNPLA3*, respectively, and (**C**,**F**) 2D depiction of *PNPLA3* interacting with the compound and the nature of forces involved in stabilizing complex of –bioactive compound WT and MT of PNPLA3, respectively. *PNPLA3*, phospholipase domain containing 3.

Molecular docking was performed to gain a deeper understanding of the binding of the bioactive compounds to the *PNPLA3* protein. The docking interactions between the selected bioactive compounds and the wild type (WT) and mutant type (MT) of the *PNPLA3* protein are shown in Table 4, Figure 1 and Figure S1–S4, respectively. The molecular docking analysis showed that the binding energy between the MT of *PNPLA3*_I148M, delphinidin-3-caffeoyl-glucoside, pyranocyanin A, delta-viniferin, kaempferol-7-glucoside, and petunidin-3-rutinoside was lower than the WT of *PNPLA3*_I148M, and they had a higher number of hydrogen bonds between *PNPLA3* and bioactive compounds than others. The more hydrogen bonds the compound has, the stronger and more stable the binding with *PNPLA3* [24]. The binding affinity of *PNPLA3* and the bioactive compounds comprised not only hydrogen bonds but also Van der Waals, alkyl, and carbon–hydrogen bonds. It indicated that the binding affinity between the MT of *PNPLA3*_I148M and the five compounds was higher than with the WT, and the inhibitory effect of the five compounds on the MT of *PNPLA3*_I148M was stronger than that on the WT.

### 3.5. Molecular Dynamics (MD) Simulation

**Figure 2:**
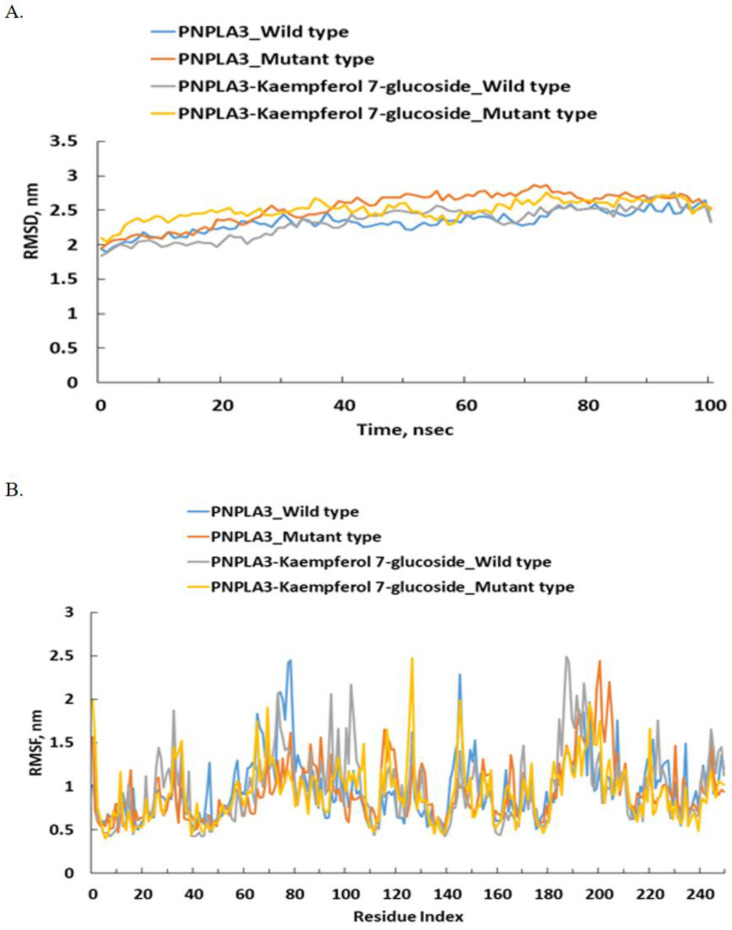
Molecular dynamics (MD) simulation of *PNPLA3* I148 (WT; rs738409) and 148M (MT) and kaempferol-7-glucoside interaction. (**A**) Variation in RMSD (root mean square deviation) of *PNPLA3* alone and *PNPLA3*–compound complex as a function of simulation. (**B**) RMSF (root mean square fluctuation) in *PNPLA3* in the absence and presence of the compound. *PNPLA3*, phospholipase domain containing 3; WT, wild type; MT, mutated type.

The RMSD and RMSF were calculated using 100 ns simulations to investigate the stability and dynamics of well-bound ligand–protein complexes with the WT and MT of *PNPLA3*_I148M (Figure 2 and Figures S5–S8). It was observed that the lower the RMSD, the higher the stability of the protein. The RMSD of kaempferol-7-glucose, petunidin-3-rutinoside, delphinidin-3-caffeoyl-glucoside, and delta-viniferin in the WT of *PNPLA3*_I148M was lower than that of the MT, while pyranocyanin A showed the opposite trend (Figure 2 and Figures S5–S8). However, the RMSD ranged between approximately 1.75 and 3.5 nm within 100 s of the wild-type and mutant *PNPLA3*_I148M. The RMSF of the five selected bioactive compounds in the WT and MT *PNPLA3* fluctuated between 0.5 and 3 nm (Figure 2 and Figures S5–S8).

### 3.6. HepG2 Cell Viability

**Figure 3:**
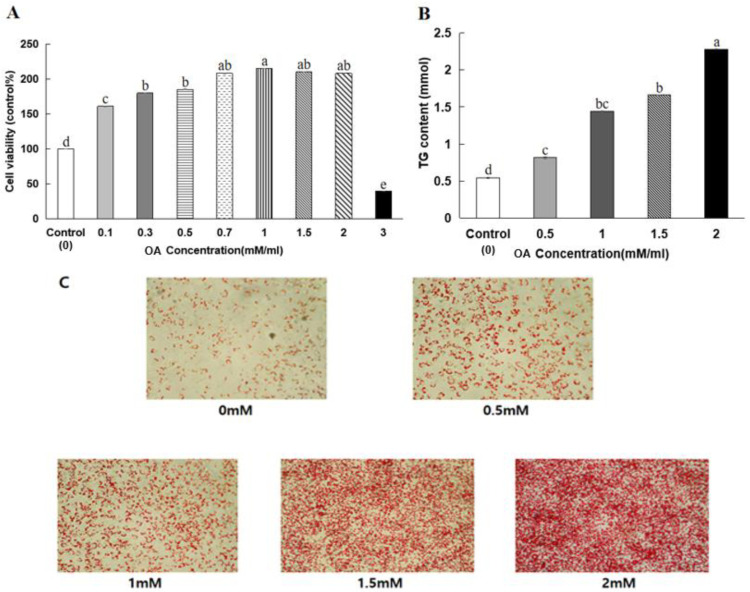
Oleic acid (OA) induces steatosis in HepG2 cells. (**A**) The viability assay of HepG2 cells was treated with different concentrations of oleic acid (OA) for 48 h. (**B**) Measurement of triglyceride content in HepG2 cells after incubation with 2 mM OA for 48 h. (**C**) Oil Red O staining to detect intracellular lipid droplets in HepG2 cells treated with 0, 0.5, 1, 1.5, and 2 mM OA for 48 h. a–e Different letters indicated significant difference between the groups by Tukey test at *p* < 0.05.

**Figure 4:**
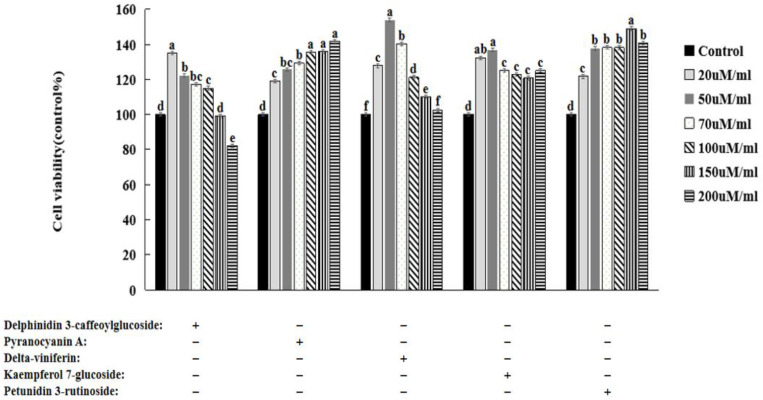
Changes in HepG2 cell viability after treatment with bioactive compounds. a–f Different letters indicated significant difference between the groups by Tukey test at *p* < 0.05.

As seen in Figure 3, treatment with OA at concentrations below 2 mM did not affect cell viability after 48 h of incubation. However, when the OA concentration was >2 mM, the viability of the HepG2 cells significantly decreased (*p* < 0.05). Therefore, 2 mM OA was used in the follow-up studies. Oil Red O staining was used to observe the lipid droplet formation with the OA treatment of the HepG2 cells. After treating the HepG2 cells with 2 mM OA for 48 h, many lipid droplets were formed in the cells (*p* < 0.01). Consistent with the Oil Red O staining results, the triglyceride content in the HepG2 cells significantly increased after the 2 mM OA treatment (Figure 3). It suggested that 2 mM of OA could induce lipid accumulation and lipid droplet formation without impacting cell viability in HepG2 cells. At concentrations below 150 μg/mL, delphinidin-3-caffeoyl-glucoside, pyranocyanin A, delta-viniferin, kaempferol-7-glucoside, and petunidin-3-rutinoside did not affect the viability of HepG2 cells (Figure 4).

### 3.7. Lipid Peroxide Contents in HeG2 Cells

**Figure 5:**
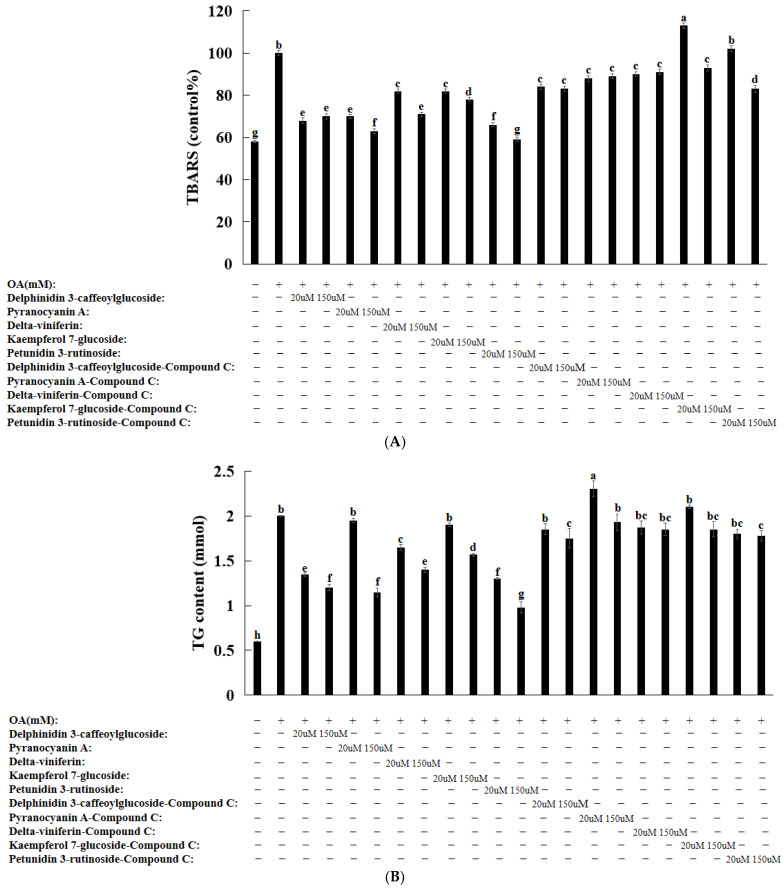
Lipid levels in HepG2 cells after treatment with oleic acid and compounds. (**A**) Lipid peroxide contents. (**B**) Triglyceride contents. a–h Different letters indicated significant difference between the groups by Tukey test at *p* < 0.05.

The malondialdehyde contents representing lipid peroxides were measured using the thiobarbituric acid reactive substance (TBARS) quantification method. Compared to no OA, lipid peroxidation significantly increased after the OA treatment, whereas the bioactive compound treatment significantly decreased lipid peroxidation. A high dosage of petunidin 3-rutinoside treatment decreased lipid peroxide contents as much as that seen without OA treatment. The compound C pretreatment suppressed the decrease in lipid peroxide contents due to the treatment with natural compounds. These results indicated that the bioactive compounds potentially exhibited their antioxidant effect through the AMPK pathways in the OA-treated cells (Figure 5A).

### 3.8. Natural Compounds in HepG2 Cells Induced by Oleic Acid

**Figure 6:**
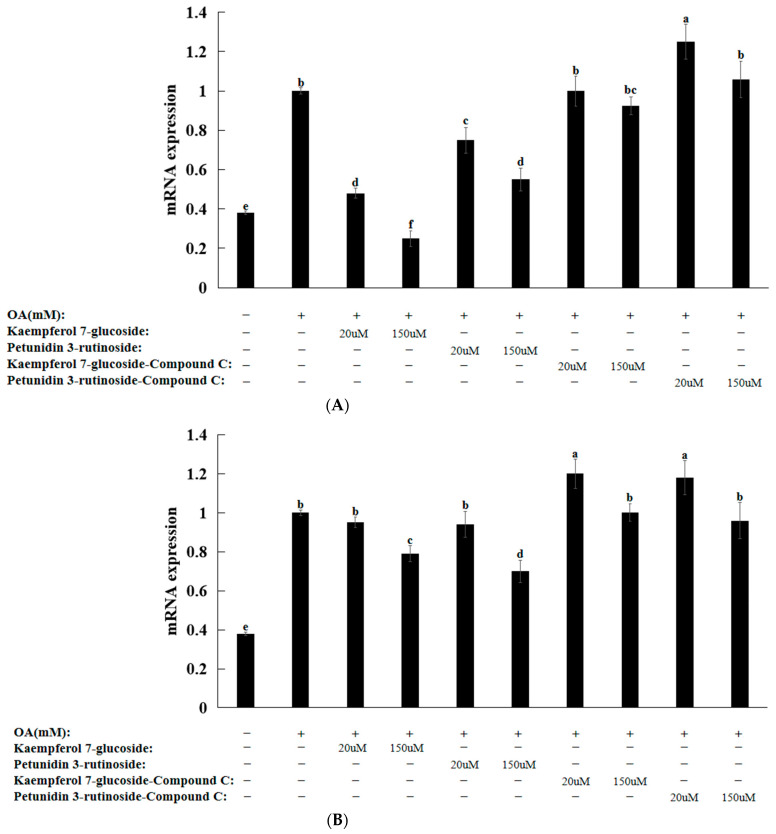
mRNA expression of lipid-metabolism-related genes after pretreatment with compound C and treatment with kaempferol 7-glucoside and petunidin 3-rutinoside. (**A**) *PNPLA3* (**B**) *SREBP-1c PNPLA3*: patatin-like phospholipase domain-containing protein 3; *SREBP-1c*: Sterol regulatory element binding protein. a–f Different letters indicated significant difference between the groups by Tukey test at *p* < 0.05.

The OA treatment of HepG2 cells induced a morphology similar to hepatic steatosis [25,26,27]. Treatment with OA alone significantly increased the amounts of intracellular triglycerides. However, the triglyceride contents significantly decreased after treatment with the bioactive compound (Figure 5B). However, pretreatment with compound C blocked its inhibitory effect on cellular triglyceride levels (Figure 5B). The OA treatment increased the mRNA levels of *SREBP-1c* and *PNPLA3*. However, kaempferol 7-glucoside and petunidin 3-rutinoside effectively prevented the increase in mRNA levels (Figure 6). However, pretreatment with compound C inhibited and reversed the bioactive-compound-mediated effects (Figure 6). Collectively, these findings imply that AMPK is involved in adipogenesis gene expression and OA-induced intracellular triglyceride accumulation.

## 4. Discussion

Recent epidemiological projections suggest that the incidence of NAFLD and NASH is likely to increase rapidly in the coming years due to the influence of urbanization [28,29,30]. This rise is anticipated to impose substantial clinical and economic burdens on the healthcare system in the coming years. Typically, patients with NAFLD and NASH do not exhibit severe symptoms, and their diagnosis usually relies on ultrasound or a liver biopsy [31,32]. Identifying individuals with potential genetic predispositions at an early age is crucial for preventing the development of NAFLD. Across various countries and ethnicities, the *PNPLA3*_rs738409 polymorphism has been identified as exerting the most significant impact on NAFLD [33,34]. The present study aimed to investigate the impact of the *PNPLA3*_rs738409 polymorphism on NAFLD risk and to explore the interaction between genetic and lifestyle factors on NAFLD risk in participants aged over 45 years. The results may contribute to developing effective strategies for its prevention and management through in silico analysis, cell-based studies, and human studies.

Evidence suggests that the protein expressed by the *PNPLA3* (WT) gene exists in lipid droplet membranes and is responsible for the postprandial remodeling of lipid droplets through its triglyceride hydrolase activity [35,36]. The protein variant *PNPLA3* (I148M) reduces hydrolase activity. It has also been shown to promote the production of profibrotic cytokines (including CCL2 and CCL5), which activate hepatic stellate cells (HSC) and promote inflammation and fibrosis in NAFLD/NASH [37,38]. The present study showed that *PNPLA3* (I148M) elevated NAFLD risk, which might be linked to increased *PNPLA3* gene expression. Therefore, reducing the expression of the *PNPLA3* gene by consuming bioactive compounds may have therapeutic benefits for NAFLD patients with risk alleles [39].

*PNPLA3* is a direct target gene of *SREBP-1c*, a key transcription factor that primarily regulates the expression of critical adipose genes of fatty acid synthase (FAS) and acetyl CoA carboxylase 1 (ACC1), and its activity is controlled by AMPK [40,41]. In the present study, *PNPLA3* modulators altered fat accumulation by suppressing AMPK. The increase in liver adipogenesis in NAFLD patients is attributed to *SREBP-1c* activation. As a result, decreasing *SREBP-1* activity is likely to diminish *PNPLA3* expression and ameliorate related steatosis. In this process, AMPK controls *SREBP-1c* to decrease fat deposition. A recent study found that the high expression of human *PNPLA3* (I148M) in the livers of transgenic mice boosted *SREBP-1c* mRNA expression [42]. Furthermore, no evidence has been found to support the regulatory role of *PNPLA3* (I148M) on AMPK, and the mechanism by which *PNPLA3* regulates AMPK needs to be clarified. The present study showed that *SREBP-1c* and *PNPLA3* gene expression increased in OA-treated HepG2 cells, and the AMPK inhibitor further elevated their gene expression. Therefore, it was shown that AMPK controls *PNPLA3* expression through *SREBP-1c*.

The I148M polymorphism of *PNPLA3* (rs738409, C>G) is associated with the occurrence and progression of NAFLD. However, despite significant research in in vitro and in vivo models, the mechanism by which *PNPLA3* (I148M) induces hepatic steatosis is unclear. In vitro investigations have demonstrated that the I148M variant restricts substrate access to the active site of the lipase enzyme, resulting in loss of lipase activity and steatosis [43]. *PNPLA3* (148M) has also been reported to induce hepatic steatosis through the accumulation of *PNPLA3*. Reduction in *PNPLA3* levels by short hairpin RNA (shRNA) knockdown has been shown to reduce liver triglyceride content in mice overexpressing *PNPLA3* (148M) [42]. However, in the livers of mice, neither inactivation nor overexpression of *PNPLA3* resulted in steatosis [42,44]. Therefore, the *PNPLA3* (148M) variant is associated with the accumulation of *PNPLA3*, leading to steatosis. Therapies that lower *PNPLA3* levels can help correct hepatic steatosis in people with the *PNPLA3*(148M) variation [36].

Currently, Rezdiffra (resmetirom) is the first medication approved by the FDA for the treatment of NAFLD. Rezdiffra is a thyroid hormone receptor (THR)-β agonist that acts primarily in the liver. Thyroid hormones, thyroxine, and its active derivative, triiodothyronine, are primary regulators of lipid metabolism, primarily through activation of the THR-β isoform in hepatocytes [45]. Activation of THR-β increases cholesterol metabolism through the expression of the CYP7A1 enzyme and reduces de novo lipogenesis (DNL) through inhibition of *SREBP-1* expression [46]. These activities suggest that Rezdiffra may provide therapeutic benefits in patients with dyslipidemia and increased DNL, which are key features of NAFLD. The accumulation of lipotoxic lipids in the liver is a major driver of the development of NASH and fibrosis. Therefore, drugs that can suppress DNL may represent a promising future approach for NAFLD treatment. The present study showed that *PNPLA3* inhibitors can decrease DNL by potentiating AMPK activity and decreasing *SREBP-1c* expression, further highlighting the potential of targeting lipid metabolism pathways for NAFLD management.

Several plant products and compounds derived from these plants have been proven to benefit liver health. The present study investigated whether bioactive compounds have a protective effect on NAFLD and whether this protective effect comes from regulating energy homeostasis and the AMPK/autophagy signaling pathways linked to *PNPLA3*. We used an AMPK inhibitor (compound C) instead of gene mutation models. The use of inhibitors has several advantages. The inhibitors only need to be added to the culture medium or directly injected into animals and can act on a wide range of cell types. In terms of time, the effect of inhibitors can be achieved relatively quickly, and the dosage and time can be flexibly adjusted. Therefore, inhibitors are relatively simple, flexible, and suitable for many target cell types. We used compound C, an AMPK inhibitor, to validate the effect of AMPK inactivation on the OA-induced activation of HepG2 cells. Compound C inhibits the activation of AMPK. Inactivation of AMPK by compound C resulted in the increased expression of *SREBP-1c* and *PNPLA3*. Our results in HepG2 cells are consistent with the Xu et al. study [47]. These results showed that compound C reversed the flavonoid effects and confirmed the involvement of the flavonoids in the AMPK/autophagy pathway and inhibition of steatosis activated by oleic acid. Increased lipogenesis contributes to hepatic steatosis, and *SREBP-1c* is a transcription factor involved in hepatic lipogenesis. *PNPLA3*, an *SREBP-1c* target gene, is also implicated in lipogenesis. The inhibition of *SREBP-1C* and *PNPLA3* can help reduce hepatic fat accumulation. Treatment with the bioactive compounds, including kaempferol-7-glucoside and petunidin-3-rutinoside, significantly decreased the expression of *SREBP-1c* and *PNPLA3* in oleic acid-treated cells, as well as the malondialdehyde levels, indicating that the bioactive compounds have antioxidant characteristics.

While this study yields valuable insights, several critical limitations merit consideration. First, the study exclusively examined middle-aged and elderly populations, necessitating further investigation into the relationship between *PNPLA3*_rs738409, liver fat content, and metabolic disorders among adolescents and young individuals. Second, the generalizability of our findings across diverse racial groups requires validation. Third, due to the cross-sectional nature of our data derived from a sizable cohort, establishing causal relationships and evaluating the prognostic relevance of *PNPLA3*_rs738409 to NFALD remains infeasible. Prospective studies are essential for addressing these inquiries. Furthermore, despite conducting cell experiments for validation, there needs to be more cell validation for mutated genes and animal experiments that corroborate our findings. Conversely, this study exhibited several notable strengths, including a substantial participant cohort, integration of lifestyle data, a targeted genetic focus, validation through cell-based and in silico methods, clinical relevance, and the potential to contribute to future research. These attributes collectively enhance our comprehension of the intricate interplay between *PNPLA3* rs738409, lifestyle factors, and NAFLD, providing the potential for more efficacious diagnostic and therapeutic approaches for the condition.

## 5. Conclusions

*PNPLA3*_rs738409 interacted with the PBD, WSD, and smoking status in a large cohort of middle-aged and elderly individuals. For the persons with the *PNPLA3*_rs738409G risk allele, it was better to have a PBD and avoid a WSD and smoking. Notably, specific bioactive compounds, such as delphinidin-3-caffeoyl-glucoside, pyranocyanin A, delta-viniferin, kaempferol-7-glucose, and petunidin-3-rutinoside, exhibited a propensity to lower binding energy with *PNPLA3* I148M in molecular docking analysis. Furthermore, kaempferol-7-glucose and petunidin-3-rutinoside effectively mitigated oleic acid-induced lipid droplet formation and triglyceride levels in HepG2 cells, operating through the AMPK/*SREBP-1C*/*PNPLA3* pathway. Future research should focus on further elucidating the causative relationships between smoking, PBDs, WSDs, and NAFLD, as well as exploring the potential of the identified bioactive compounds as therapeutic agents for individuals with the *PNPLA3*_rs738409G risk allele.

## Figures and Tables

**Table 1:** Participants’ socioeconomic level and metabolic characteristics in NAFLD.

	Non-NAFLD (*n* = 48,999)	NAFLD (*n* = 2089)	Adjusted OR (95% CI)
Age (years) ^1^	53.77 ± 8.033	54.90 ± 7.886 ***	1.040 (0.945~1.144)
Genders (men: N, %)	14,908 (30.4)	918 (43.9) ***	1.343 (1.176~1.535) ***
BMI (kg/m^2^) ^2^	23.65 ± 2.775	25.56 ± 3.403 ***	2.581 (2.357~2.827) ***
Waist circumference ^3^	79.93 ± 8.35	85.66 ± 9.26 ***	1.479 (1.304~1.678) ***
Plasma total cholesterol (mg/dL) ^4^	197 ± 35.22	200 ± 39.97 **	1.259 (1.126~1.408) ***
Plasma triglyceride (mg/dL) ^5^	119 ± 76.22	161 ± 126 ***	1.831 (1.635~2.049) ***
Hypertension (N, %) ^6^	12,997 (26.5)	849 (40.7) ***	1.317 (1.192~1.456) ***
Type 2 diabetes (N, %) ^7^	3641 (7.4)	407 (19.5) ***	2.330 (2.062~2.633) ***
Education (N, %) ^8^			
<High school	14,907 (30.7)	703 (33.9) **	
High school	21,027 (43.3)	837 (40.4)	1.021 (0.910~1.146)
College more	12,616 (26.0)	534 (25.7)	1.002 (0.869~1.155)
Income (N, %) ^9^			
<$2000/month	14,379 (31.1)	656 (33.4)	
$2000–4000/month	27,969 (60.5)	1152 (58.7)	0.996 (0.891~1.113)
>$4000/month	3888 (8.4)	154 (7.8)	0.876 (0.694~1.106)
Energy intake (EER %) ^10^	98.80 ± 31.66	99.01 ± 32.59	1.093 (0.986~1.211)
CHO (EER %) ^11^	71.79 ± 6.93	71.80 ± 7.23	1.029 (0.936~1.131)
Protein (EER %) ^12^	13.39 ± 2.56	13.45 ± 2.65	1.033 (0.942~1.133)
Fat (EER %) ^13^	13.83 ± 5.38	13.72 ± 5.56	1.077 (0.938~1.237)
Cholesterol intake ^14^	169 ± 124	174 ± 138	1.083 (0.951~1.233)
Na intake (mg) ^15^	2421 ± 1364	2545 ± 1496 ***	1.001 (0.896~1.118)
Fiber intake(g) ^16^	14.61 ± 9.26	15.45 ± 10.51 ***	1.045 (0.936~1.166)
Alcohol (g) ^17^	2.09 ± 4.77	3.37 ± 6.35 ***	1.406 (1.116~1.770) **
KBD (%) ^18^	15,710 (32.1)	756 (36.2) ***	1.039 (0.938~1.150)
PBD (%) ^18^	16,963 (34.6)	638 (30.5) ***	0.948 (0.851~1.056)
WSD (%) ^18^	19,151 (39.1)	859 (41.1)	0.940 (0.847~1.043)
RMD (%) ^18^	16,276 (33.2)	673 (32.2)	1.014 (0.916~1.121)
Smoking (Number, %)	11,458 (23.5)	717 (34.4) ***	1.167 (1.013~1.345) *

Values represent adjusted means ± standard deviation or the number (N) of participants and percentage. OR, odds ratio; CI, confidence intervals; KBD, Korean balanced diet; PBD, plant-based diet; WSD, Western-style diet; RMD, rice-main diet. The cutoff points of the reference were as follows: ^1^ 55 years old for age, ^2^ 25 kg/m^2^ BMI, ^3^ 90 cm for men and 85 cm for women waist circumferences, ^4^ 230 mg/dL plasma total cholesterol concentrations, ^5^ 200 mg/dL plasma triglyceride concentrations, ^6^ 140 mmHg systolic blood pressure (SBP), 90 mmHg diastolic blood pressure (DBP) plus hypertension medication, ^7^ 126 mL/dL fasting serum glucose plus diabetic drug intake, ^8^ high school graduation and ^9^ $2000/month income, ^10^ <estimated energy requirement (EER), ^11^ 72 energy percent (En%) for carbohydrate (CHO), ^12^ 13 En% for protein, ^13^ 20 En% for fat, ^14^ 250 mg/day cholesterol intake, ^15^ 1600 mg for sodium, ^16^ 20 g for fiber intake, ^17^ 20 g for alcohol, ^18^ 75th percentiles of each dietary pattern. Covariates for adjusted means and OR: age, gender, BMI, residence area, physical activity, education, smoking, and energy intake in ANCOVA and logistic regression models, respectively. * Significant differences by NAFLD at *p* < 0.05, ** at *p* < 0.01, *** *p* < 0.001.

**Table 2:** Association analysis of *PNPLA3* I148M to influence NAFLD.

CHR	SNP	Position	Mi	Ma	OR	Adjust *p* Value	MAF	HWE_P	GENE	Function
22	rs738409	44324727	G	C	1.487	1.482 × 10^−33^	0.417	0.819	*PNPLA3*	Missense variant

*PNPLA3*, patatin-like phospholipase domain-containing protein 3; CHR, chromosome; SNP, single-nucleotide polymorphism; Mi, minor allele; Ma, major allele; OR, odds ratios for NAFLD in the reference of the major allele; adjusted *p* value, *p* value for OR adjusted for age, gender, body mass index, residence area, energy intake, physical activity, smoking status, alcohol intake, and education; MAF, minor allele frequency; HWE_P, *p*-value for Hardy–Weinberg equilibrium.

**Table 3:** After adjusting the odds ratio of NAFLD risk, rs738409 risk score according to lifestyle pattern.

	Major (*n* = 17,448)	Hetero (*n* = 24,821)	Minor (*n* = 8819)	Gene–Nutrient Interaction *p* Value
Low energy ^1^ High energy	1	1.330 (1.167~1.516) 1.360 (1.109~1.669)	2.342 (2.019~2.716) 1.941 (1.524~2.473)	0.218
Non-smoke Former + current smokers	1	1.310 (1.148~1.495) 1.386 (1.139~1.686)	1.892 (1.619~2.210) 3.121 (2.519~3.867)	<0.0001
Low KBD ^2^ High KBD	1	1.355 (1.181~1.556) 1.308 (1.090~1.570)	2.206 (1.883~2.584) 2.247 (1.823~2.771)	0.360
Low PBD ^2^ High PBD	1	1.367 (1.197~1.562) 1.285 (1.056~1.564)	2.404 (2.066~2.798) 1.896 (1.509~2.383)	0.019
Low WSD ^2^ High WSD	1	1.256 (1.091~1.446) 1.475 (1.235~1.760)	1.955 (1.660~2.304) 2.696 (2.208~3.291)	0.017
Low RMD ^2^ High RMD	1	1.388 (1.214~1.588) 1.236 (1.020~1.497)	2.299 (1.970~2.682) 2.050 (1.644~2.555)	0.462
No exercise ^3^ Exercise	1	1.348 (1.150~1.580) 1.329 (1.141~1.549)	2.394 (2.000~2.865) 2.070 (1.732~2.473)	0.072

Values represent odd ratios and 95% confidence intervals. The cutoff points were as follows: ^1^ <Estimated energy requirement defined in dietary reference index, ^2^ <75th percentiles; ^3^ <moderate exercise for 150 min/day; multiple logistic regression models include the corresponding main effects, interaction terms of SNPs and main effects (energy and nutrient intake), and potential confounders such as age, gender, BMI, residence area, physical activity, education, smoking, and energy intake. Reference was the major.

**Table 4:** The intermolecular binding energy between bioactive compounds and *PNPLA3* active site.

Compound Name	Effective Food	Residues Involved in Hydrogen Bond		Residues Involved in Hydrophobic Interactions		Wild Type	Mutant Type
		Wild-Type	Mutant-Type	Wild-Type	Mutant-Type	Docking Energy, ΔG (kcal mol^−1^)	
Delphinidin 3-caffeoyl-glucoside	grape	His204, Tyr188, Ser152, Cys146, Arg74	Tyr188, Thr200, Ser199, Ser47, Asp166, Arg74	Leu72, Leu51, Pro229, Phe150, Ile148	Phe150, Leu203, Met148, Leu154	−7.6	−9.4
Pyranocyanin A	blackcurrant	Arg74, Ser152, Val197, Ser199, Lys198, Pro195, Tyr191	Val197, Lys198, Ser199, Pro195, Asn201, Tyr188, His204, Cys146	His204, Phe150, Pro228, Pro229	Cys15, Met148, Lys198, Tyr188, Phe150, Pro229	−9.1	−10.2
Delta-viniferin	grape	His204, Pro229, Asn201, Pro195	Cys15, Cys146, Arg74, Ser152, Ser199	Pro228, Phe150, Tyr191	Met148, Pro228, Phe150, Pro229	−9	−10
Kaempferol 7-glucoside	flaxseed	Asp166, Ser152, Asn201, Ser199	Cys146, Arg74, Ser152, Tyr191, Pro195, Asn201, His204	Cys15, Tyr188, Ile148, Pro229, Phe150, Pro228	Met148, Phe150, Pro228, Pro229	−8.2	−9.6
Petunidin 3-rutinoside	mulberry	His204, Asn201, Ser199, Lys198	Ser199, Asn201, Tyr191, Arg74, Pro228, Pro22, Ser78, Pro195, Ser152	Phe150, Tyr191, Pro195	Tyr191, Leu154, Met148	−7.9	−9

## Data Availability

The data was deposited in the Korean biobank (Osong, Republic of Korea) and provided for the research upon request.

## Supplementary Materials

The following supporting information can be downloaded at: https://www.mdpi.com/article/10.3390/nu16081239/s1. Table S1. List of primers of *PNPLA3* and *SREBP-1c* genes; Figures S1–S4. Molecular docking of *PNPLA3*_rs738409 (I148) and MT (148M) with a bioactive compound; Figures S5–S8. Molecular dynamics (MD) simulation of *PNPLA3*_rs738409 (I148) and MT (148M) and bioactive compound interaction.
