# Supplementary material for: The Role of PNPLA3_rs738409 Gene Variant, Lifestyle Factors, and Bioactive Compounds in Nonalcoholic Fatty Liver Disease: A Population-Based and Molecular Approach towards Healthy Nutrition
Source: Nutrients. 2024 Apr 21;16(8):1239. doi: 10.3390/nu16081239 (PMC11054963; doi:10.3390/nu16081239)
Supplement: Supplementary file 1 [file nutrients-16-01239-s001.zip › nutrients-2969796-supplementary.pdf]

Figure S1. Molecular docking of *PNPLA3*_rs738409 (I148) and MT (148M) with a bioactive compound for delphinidin 3-caffeoylglucoside

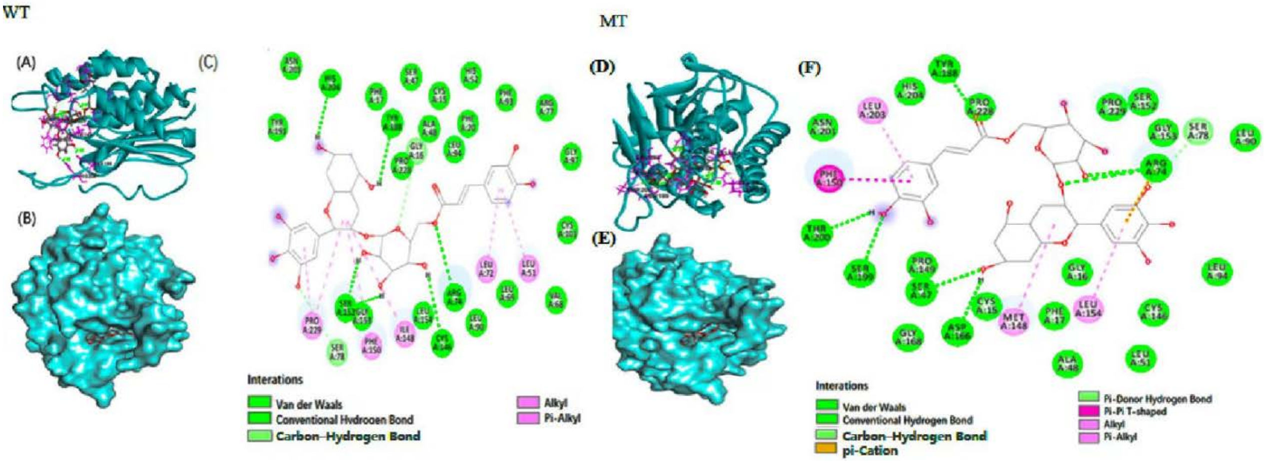

(A,D) Diagrammatic representation of the compound (ball and stick model) binding with WT and MT of *PNPLA3*, respectively. (B,E) binding of the compound at the central cavity with WT and MT of *PNPLA3*, respectively. (C,F) 2D depiction of *PNPLA3* interacting with the compound and the nature of forces involved in stabilizing–complex of bioactive compound WT and MT of *PNPLA3*, respectively. *PNPLA3*, phospholipase domain containing 3.

Figure S2. Molecular docking of *PNPLA3*_rs738409 (I148) and MT (148M) with a bioactive compound for pyranocyanin A

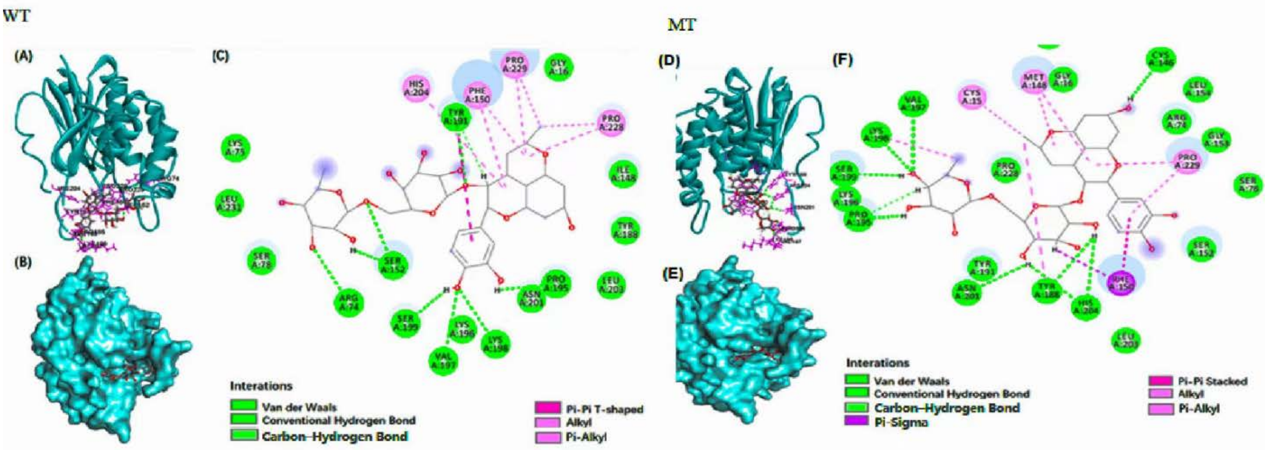

(A,D) Diagrammatic representation of the compound (ball and stick model) binding with WT and MT of *PNPLA3*, respectively. (B,E) binding of the compound at the central cavity with WT and MT of *PNPLA3*, respectively. (C,F) 2D depiction of *PNPLA3* interacting with the compound and the nature of forces involved in stabilizing–complex of bioactive compound WT and MT of *PNPLA3*, respectively. *PNPLA3*, phospholipase domain containing 3.

Figure S3. Molecular docking of *PNPLA3*_rs738409 (I148) and MT (148M) with a bioactive compound for delta-viniferin

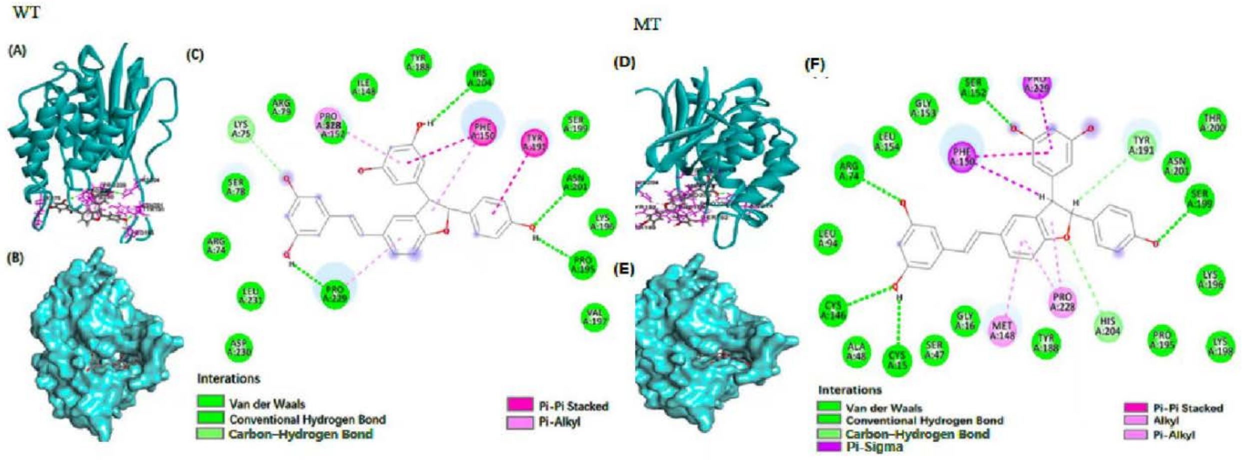

(A,D) Diagrammatic representation of the compound (ball and stick model) binding with WT and MT of *PNPLA3*, respectively. (B,E) binding of the compound at the central cavity with WT and MT of *PNPLA3*, respectively. (C,F) 2D depiction of *PNPLA3* interacting with the compound and the nature of forces involved in stabilizing–complex of bioactive compound WT and MT of *PNPLA3*, respectively. *PNPLA3*, phospholipase domain containing 3.

Figure S4. Molecular docking of *PNPLA3*_rs738409 (I148) and MT (148M) with a bioactive compound for petunidin 3-rutinoside

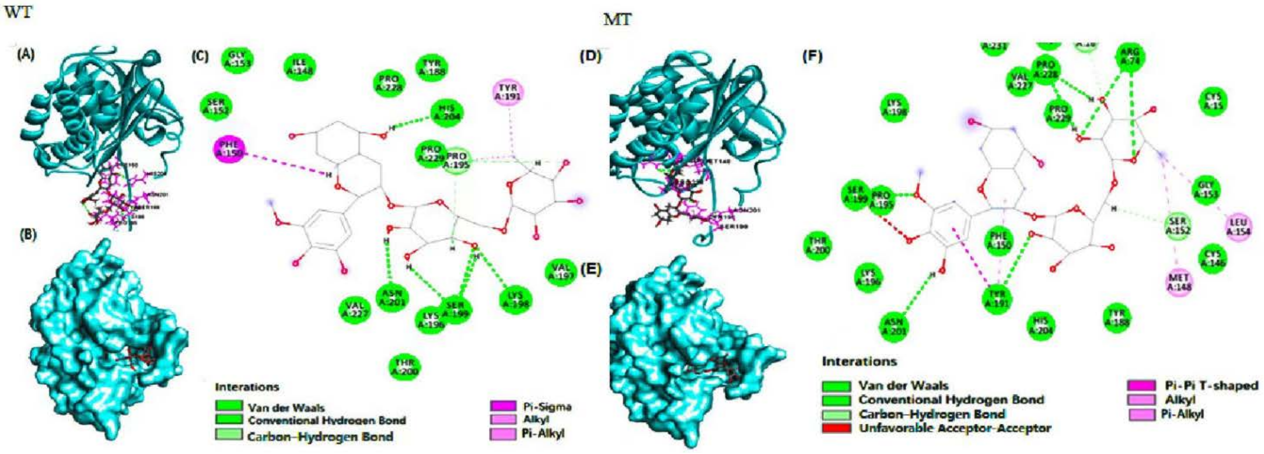

(A,D) Diagrammatic representation of the compound (ball and stick model) binding with WT and MT of *PNPLA3*, respectively. (B,E) binding of the compound at the central cavity with WT and MT of *PNPLA3*, respectively. (C,F) 2D depiction of *PNPLA3* interacting with the compound and the nature of forces involved in stabilizing–complex of bioactive compound WT and MT of *PNPLA3*, respectively. *PNPLA3*, phospholipase domain containing 3.

Figure S5. Molecular dynamics (MD) simulation of *PNPLA3*_rs738409 (I148) and MT (148M) and delphinidin 3-caffeoylglucoside interaction.

A. RMSD (root mean square deviation)

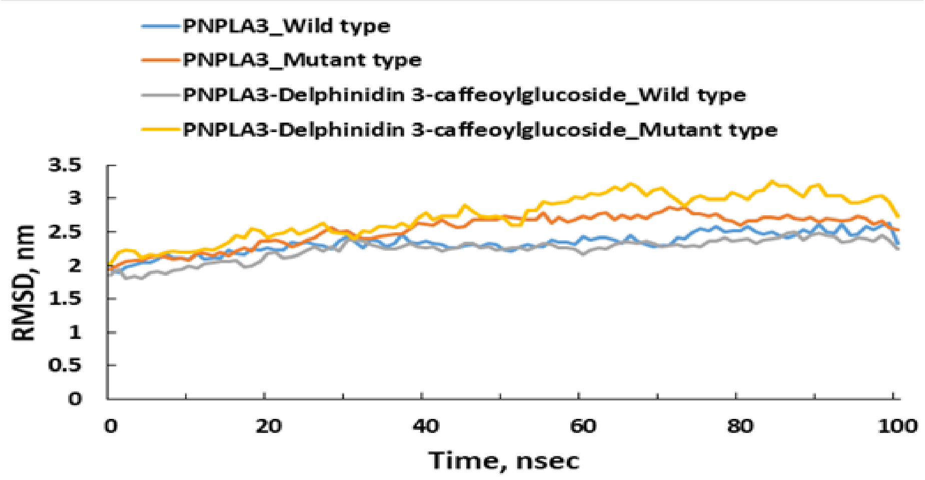

B. RMSF (root mean square fluctuation)

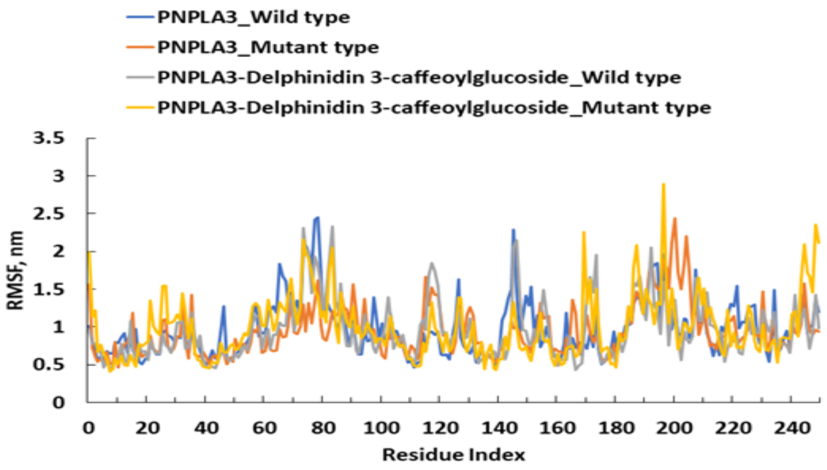

Figure S6. Molecular dynamics (MD) simulation of *PNPLA3*_rs738409 (I148) and MT (148M) and pyranocyanin A interaction.

A. **RMSD (**root mean square deviation)

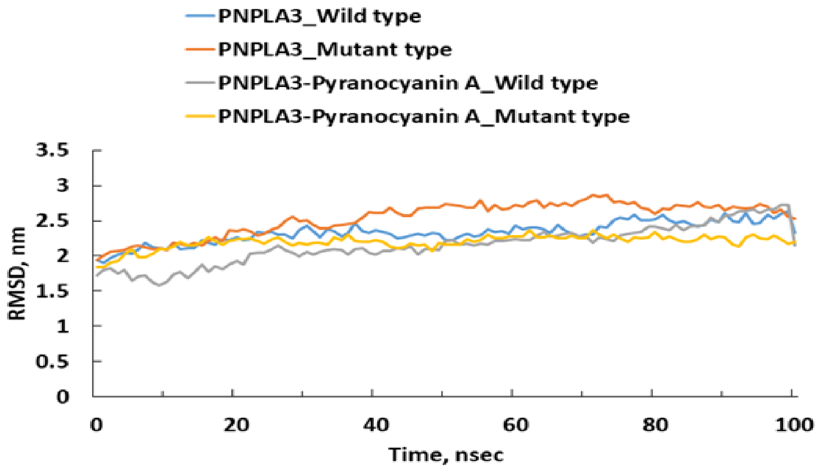

B. RMSF (root mean square fluctuation)

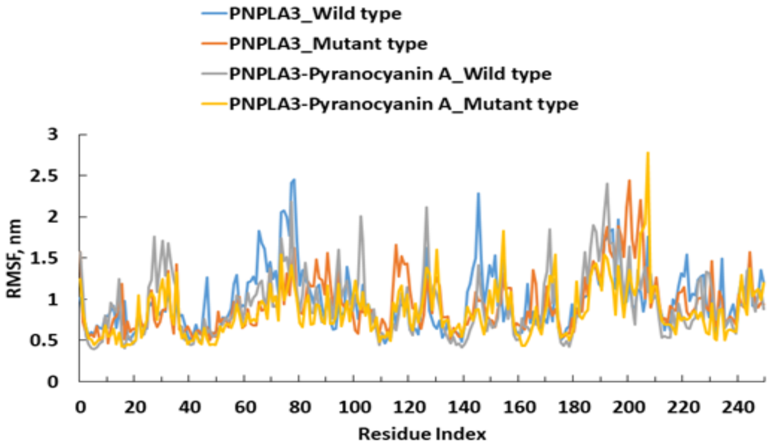

Figure S7. Molecular dynamics (MD) simulation of *PNPLA3*_rs738409 (I148) and MT (148M) and delta-viniferin interaction.

A. RMSD (root mean square deviation)

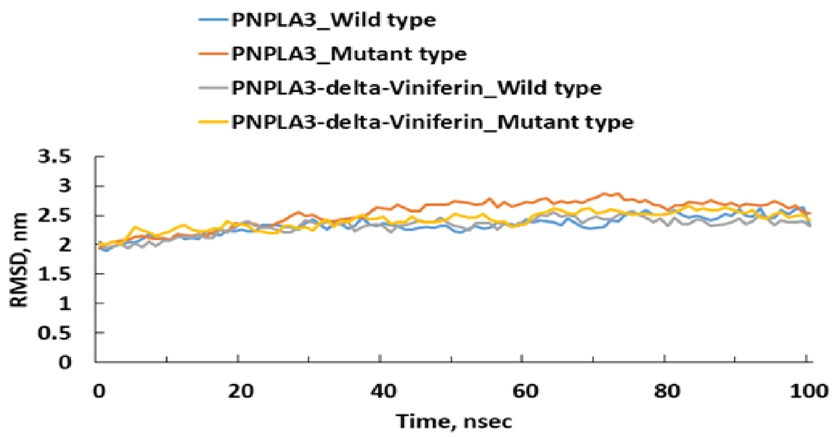

B. RMSF (root mean square fluctuation)

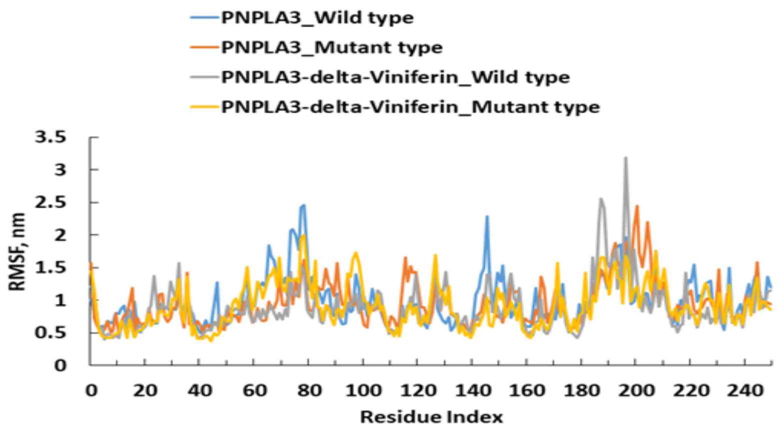

Figure S8. Molecular dynamics (MD) simulation of *PNPLA3*_rs738409 (I148) and MT (148M) and petunidin 3-rutinoside interaction.

A. RMSD (root mean square deviation)

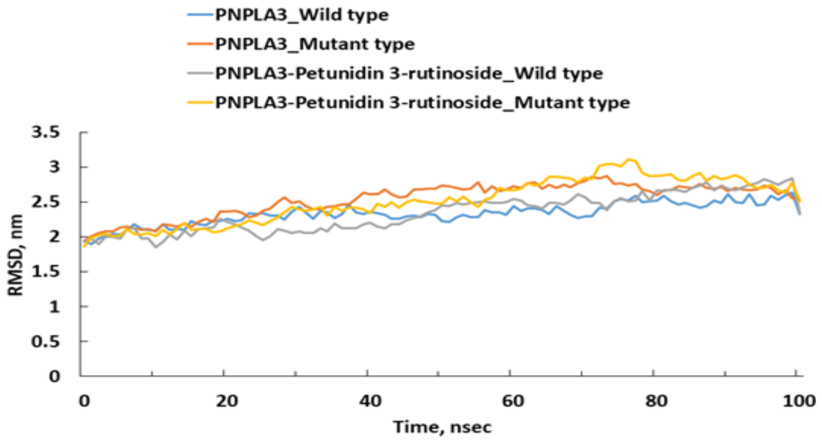

B. RMSF (root mean square fluctuation)

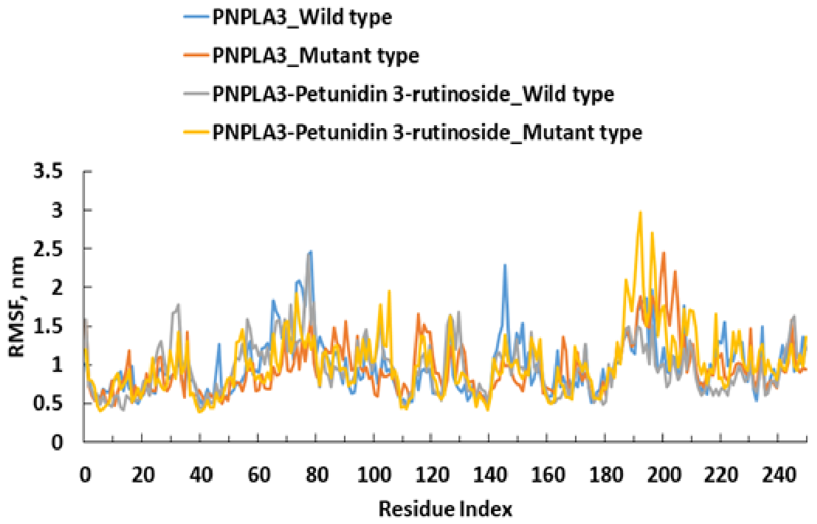

Table S1. Primer sequence used for qRT-PCR

PNPLA3	Forward:	CTGTACCCTGCCTGTGGAAT
	Reverse:	TCGAGTGAACACCTGTGAGG
SREBP-1c	Forward:	CGGAACCATCTTGGCAACAGT
	Reverse:	CGCTTCTCAATGGCGTTGT
